# Saffron: A Natural Potent Antioxidant as a Promising Anti-Obesity Drug

**DOI:** 10.3390/antiox2040293

**Published:** 2013-10-29

**Authors:** Maryam Mashmoul, Azrina Azlan, Huzwah Khaza’ai, Barakatun Nisak Mohd Yusof, Sabariah Mohd Noor

**Affiliations:** 1 Department of Nutrition and Dietetics, Faculty of Medicine and Health Sciences, Universiti Putra Malaysia, UPM Serdang, Selangor 43400, Malaysia; E-Mails: mmashmoul@yahoo.com (M.M.); bnisak@medic.upm.edu.my (B.N.M.Y.); 2 Laboratory of Halal Science Research, Halal Products Research Institute, Universiti Putra Malaysia, UPM Serdang, Selangor 43400, Malaysia; 3 Department of Biomedical Sciences, Faculty of Medicine and Health Sciences, Universiti Putra Malaysia, UPM Serdang, Selangor 43400, Malaysia; E-Mail: huzwah@medic.upm.edu.my; 4 Department of Pathology, Faculty of Medicine and Health Sciences, Universiti Putra Malaysia, UPM Serdang, Selangor 43400, Malaysia; E-Mail: sabariah@medic.upm.edu.my

**Keywords:** saffron, antioxidant, crocin, obesity, overweight, weight loss

## Abstract

Obesity is associated with various diseases, particularly diabetes, hypertension, osteoarthritis and heart disease. Research on possibilities of herbal extracts and isolated compounds from natural products for treating obesity has an upward trend. Saffron (*Crocus Sativus* L. *Iridaceae*) is a source of plant polyphenols/carotenoids, used as important spice and food colorant in different parts of the world. It has also been used in traditional medicine for treatment of different types of illnesses since ancient times. Many of these medicinal properties of saffron can be attributed to a number of its compounds such as crocetin, crocins and other substances having strong antioxidant and radical scavenger properties against a variety of radical oxygen species and pro-inflammatory cytokines. The aim of this article is to assess the potential role of saffron and its constituents in the regulation of metabolic functions, which can beneficially alter obesity pathophysiology.

## 1. Introduction

Obesity and overweight are global problems since they can lead to complications associated with human health, and they can raise the risk of many diseases such as coronary heart disease, type 2 diabetes, cancers, hypertension and dyslipidemia [1]. In the last decade, chemists, nutritionists and practitioners have been working collectively to build up innovative nutritional applications to comply with people’s needs and demands to overcome overweight and obesity problems. Two different types of obesity treatment drugs are currently available in the market, including orlistat, which reduces intestinal fat absorption through inhibition of pancreatic lipase, and sibutramine, which is an anorectic. However, they are costly and have potentially dangerous side-effects. Therefore, possibilities of herbal products for managing obesity are under intensive investigation [2,3].

A variety of natural products, including natural extracts and isolated compounds from plants, have been reported to increase body weight loss and prevent diet-induced obesity [4,5,6,7,8,9]. Among the compounds used in modern nutrition and pharmacology, antioxidants are the most significant. For their special antioxidant characteristics, carotenoids and polyphenols have attracted much interest. They are able to reduce levels of glucose, triglycerides and LDL cholesterol in blood, increase energy expenditure and fat oxidation, as well as lower body weight and adiposity [10,11]. Research results have shown that they are also capable of inhibiting enzymes related to fat metabolism, including pancreatic lipase, lipoprotein lipase and glycerophosphate dehydrogenase [12,13].

Saffron is the dried stigma of the flowers of the saffron crocus (*Crocus sativus* L. *Iridaceae*), which can be classified as a potent plant antioxidant. Numerous studies indicated the health promoting properties of saffron are attributed primarily to crocin, a unique carotenoid with powerful antioxidant capacity, which makes distinctive bright yellow color of the stigma [14,15,16,17,18,19]. According to several researchers, saffron is considered a potential therapeutic drug in clinical trials [20,21]. Recently, the application of saffron in many types of neuronal and cardiovascular disorders as well as cancer has been studied [22]. Although research about the connection between saffron compounds and body weight is not definitive yet, there are several theories that saffron has a potential to combat against overweight/obesity and related metabolic disorders owing to its high antioxidant activity and different biological properties. This paper briefly reviews the available scientific evidence regarding the role of bioactive compounds of saffron in modulation of some metabolic disorders as well as the link between antioxidants of saffron and their possible anti-obesity potential. The available literature reviewed in this paper provides proof supporting the positive role of saffron in treatment of some obesity-related metabolic disorders such as hyperlipidemia, diabetes and cardiovascular disease. However, further studies are needed in order to investigate whether saffron can be regarded as an effective medication in weight loss and obesity treatment and to analyze the molecular mechanisms involved.

## 2. Saffron Bioactive Compounds

**Figure 1:**
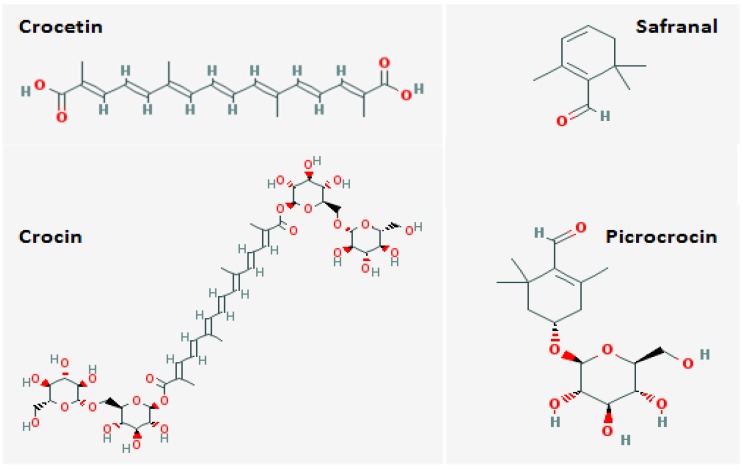
Chemical composition of the most active constituents of saffron.

The chemical composition of stigmas of *Crocus sativus* L. has been investigated in several studies during the past two decades. Reportedly, stigma of *Crocus sativus* flower contains three main metabolites; (1) Crocins which are the saffron-colored compounds (unusual water-soluble carotenoids due to their high glycosyl contents); (2) Picrocrocins which are the main substances responsible for saffron’s bitter taste; and (3) Safranal which is the volatile oil responsible for the typical saffron aroma [7,15,23]. Crocin and picrocrocin are the major compounds in saffron. Crocin is responsible for its characteristic color, and picrocrocin is a precursor of safranal. In addition to Crocin and picrocrocin, anthocyanins, flavonoids, vitamins (riboflavin and thiamine), amino acids, proteins, starch, mineral matter, gums, and other chemical compounds have been found in saffron [15,16,23]. Figure 1 illustrates the structure of the most important components of saffron.

### 2.1. General Characteristics of Crocin

Crocin (Crocetin di-gentiobiose ester) has been detected as the most prominent chemical constituent isolated from the saffron voluble spice and from the less expensive Gardenia fruit (Gardenia Jasminoides Ellis) [24,25]. Crocin (C_44_H_4_O_24_) is a di-ester which is formed from the disaccharide gentiobiose and the dicarboxylic acid crocetin and is considered as one of the few naturally occurring carotenoids easily soluble in water. Crocin has shown various pharmacological activities such as anti-oxidant, anti-cancer, learning and memory enhancer in medicinal field [8,26]. Besides its high anti-oxidant activity, the distinctive orange-red color of its pigment has made it also noteworthy for various industrial applications such as coloring matter in food and dying industry, preservative, bleaching agent for evaluation of pro-oxidant activity of foods by kinetic analysis and anti-aging agent in cosmetics [27].

### 2.2. Crocin Absorption, Bioavailability and Toxicity

Studies showed that crocin is not absorbed throughout the gastrointestinal tract. It has been observed that after oral administration of crocins, it is priorly hydrolysed to crocetin or through intestinal absorption, the absorbed crocetin is partially metabolized to mono- and di-glucuronide conjugates [28]. It was also found that oral administration of crocinis not absorbed either after single or frequent doses. Following oral administration of crocin, it is primarily excreted through the intestinal tract. Crocetin concentrations of plasma do not tend to rise with frequent oral doses of crocin and the intestinal tract function as the key site for hydrolysis of saffron [29]. Moreover, Ames study (biological assessment to determine the mutagenic potential of chemical compounds) had indicated the non-mutagenic and non-antimutagenic properties of crocin from saffron [16].

## 3. Biological Properties of Saffron

Saffron is used in natural medicine as anti-catarrhal, anti-spasmodic, nerve sedative, gingival seda-tive, diaphoretic, carminative and expectorant [15]. In a relatively recent pharmacological studies, saffron demonstrated numerous health properties such as anti-cancer and anti-toxic [16,30,31,32,33], antioxidant [14,18,21,34,35,36], antinociceptive and anti-inflammatory [37,38], anti-atherosclerosis [20,22,39], anti-diabetic and insulin resistance [40,41,42], hypotensive [43], hypolipidemic [44] and hypoglycemic [45,46,47], antidepressant and mood improving [48,49,50,51,52,53] effects. Although numerous studies have been reported in different medicinal properties of saffron and its constituents but in this paper we concentrated on scientific evidence demonstrating the potential of saffron against obesity and related metabolic disorders. Some of the major reported biological functions attributable to potential anti-obesity effect of saffron as well as experimental conditions, dosage and conclusions are summarized in Table 1.

### 3.1. Anti-Oxidant Activity of Saffron

Many studies on medicinal properties of saffron have indicated that saffron has a potent antioxidant activity which is mostly due to the presence of crocin as a unique carotenoid. The crocin bleaching assay was also designed according to this important property of crocin as a basic element for the antioxidant activity of saffron [27,54,55]. It was shown that the antioxidant properties of both methanol extract and water-methanol (50:50 v/v) extract of *Crocus sativus* stigmas were higher than those of tomatoes and carrots [27]. Kanakis *et al*. [33] reported that antioxidant activity was overall lower in saffron components than Trolox and BHT, especially safranal, but crocetin was closer to BHT and Trolox. The antioxidant activity of dimethylcrocetin was concentration dependent, with a peak in 40 μg/mL. However, the synergistic effect of all the bioactive constituents gave saffron a significant antioxidant activity. The antioxidant property of crocin was evaluated in comparable amounts with butylated hydroxyanisole (BHA) [27]. Crocin showed a high radical scavenging activity (50% and 65% for 500 and 1000 ppm solution in methanol, respectively), followed by safranal (34% for 500 ppm solution). High radical scavenging activity of these compounds is probably due to their ability to donate a hydrogen atom to the DPPH radical [21].

**Table 1:** Major biological properties of saffron attributed to potential anti-obesity effect.

Health property	Saffron/Compound	Human/animal subjects	Results	Reference
***Hypolipidemic***	Crocin	Bovine aortic endothelial cells (EC), bovine aortic smooth muscle cells (SMC) and quail	Crocin decreased OX-LDL induced EC apoptosis as well as SMC proliferation. Crocin decreased Ox-LDL and thus inhibited the formation of atherosclerosis in quails.	[22]
	Crocetin	Quails	A 9-week treatment with crocetin (25, 50, 100 mg/kg/day) reduced serum total cholesterol level and inhibited the formation of aortic plaque, reduced malonaldehyde and decreased nitric oxide in serum.	[39]
	Crocin	Rats	A 10-day treatment with crocin (25 to 100 mg/kg/day) significantly reduced serum triglyceride, total cholesterol, LDL cholesterol and VLDL cholesterol levels. The hyperlipidemic effect of crocin was attributed to its pancreatic lipase inhibition.	[44]
***Hypoglycemic & Anti-diabetic***	Crocetin	Male Wistar rats	Crocetin (40 mg/kg) prevented dexanethasone-induced insulin resistance.	[40]
	Saffron methanolic extract, crocin and safranal	Alloxan-diabetic rats	Saffron methanolic extract (80 and 240 mg/kg), crocin (50 and 150 mg/kg) and safranal (0.25 and 0.5 mL/kg) significantly reduced the fasting blood glucose and HbA1c levels and significantly increased the blood insulin levels without any significant effects on the blood SGOT, SGPT and creatinine levels in the diabetic rats compared with the control diabetic rats.	[46]
	Saffron Extract	Healthy male rats	Administration of 50 mg/kg of saffron extract for 14 days significantly decreased serum glucose, cholesterol and insulin levels.	[56]
	Crocetin	Male Wistar rats	Crocetin (40 mg/kg) improved insulin sensitivity in fructose-fed rats via normalizing the expression of both protein and mRNA of adiponectin (an insulin-sensitizing adipocytokine), TNF-α, and leptin in epididymal white adipose tissue.	[57]
***Anti-depressant***	Aqueous and ethanolic saffron extract, crocin, and safranal	Male BALB/c mice	The antidepressant activity was evaluated via forced swimming test. The aqueous and ethanolic extracts of stigma (0.2–0.8 g/kg), safranal (0.15–0.5 mL/kg) and crocin (50–600 mg/kg) reduced immobility time. Extracts, safranal, and crocin increased swimming time.	[48]
	Capsulated ethanolic saffron extract	Forty adult outpatients	In a 6-week double-blind, placebo-controlled and randomized trial, saffron extract 30 mg/day was effective in the treatment of mild to moderate depression.	[49]
	Saffron petal extract	Forty adult outpatients	In an 8-week pilot, double-blind randomized trial, petal extract (30 mg/day) was effective similar to fluoxetine (20 mg/day) in the treatment of mild to moderate depression.	[50]
	Capsulated ethanolic saffron extract	Forty adult outpatients	In a 6-week randomized and double-blind clinical trial, saffron (30 mg/day) was found to be effective similar to fluoxetine (20 mg/day) in the treatment of mild to moderate depression.	[51]
***Anti-oxidant***	Aqueous saffron extract and crocin	Rats	In crocin pretreated groups, a reduction in TBARS levels and elevation in antioxidant power (FRAP value) and total thiol as compared with control group were observed. The extract also reduced lipid peroxidation products and increased antioxidant power in ischemia-reperfusion injured rat kidney.	[58]
	Saffron extract	Rats	Liver MDA content in groups treated with 40 mg/kg saffron extract was significantly decreased as compared with that of the control group. The GSH, SOD, CAT and GSH-Px contents of the liver also significantly increased in the treatment group as compared with those in the control group.	[58]
	Crocin	Rats	Crocin dose-dependently amelio-rated collagen- and A23187-induced endogenous generation of ROS and H(2)O(2). It also abolished the H(2)O(2)-induced events of intrinsic pathway of apoptosis.	[59]
***Satiety enhancer and weight loss promoter***	Capsulated ethanolic saffron extract	Sixty overweight women	Subjects were given 1 capsule of *Satiereal* (176.5 mg/day) or an inactive placebo with no limitation in dietary intake. After 2 months, the subjects using the saffron extract reported a decrease in snacking and lost more weight than the control group.	[60]

Crocin scavenges free radicals, mainly the superoxide anions, and so may defend cells against oxidative stress. Research results have shown that crocin is beneficial for sperm cryo-conservation; therefore, it could be helpful in treatment of neurodegenerative disorders due to its great antioxidant activity [29]. Crocetin decreased lipid peroxidation induced by reactive oxygen species (ROS) in rat primary hepatocytes [61] and by BαP in mice [62]. Crocetin decreased formation of malondialdehyde (MDA) as an index of lipid peroxidation induced by ROS [62,63]. The chemopreventive property of saffron via modulation of antioxidants, lipid peroxidation, and detoxification systems was also proposed [64].

### 3.2. Hypolipidemic Effect of Saffron

Crocin has been reported as an effective hypolipidemic agent in several human and animal studies. Crocin decreased the amount of cholesterol in hyperlipidemic rats with 2 months feeding of excessive cholesterol [44]. In another study, it was found that crocin has strong triglyceridemic and cholesterolemic lowering effects in quails with coronary artery disease [22]. Further research verified that crocetin could reduce the levels of serum, total cholesterol and malondialdehyde and prevent reduction of nitric oxide in serum of hyperlipidemic-diet quails [39]. In the elucidation of the hypolipidemic mechanism of crocin, Sheng *et al*. [44] indicated that crocin inhibited the absorption of dietary fat and cholesterol. They reported this inhibition was very much related to the hydrolysis of fat. Likewise, the modified fat-balance method indicated that crocin increased excretion of fecal fat and cholesterol in rats, but it had no impact on the elimination of bile acids. Data of the in situ loop method and enzyme assay demonstrated that crocin could not directly inhibit the absorption of cholesterol from rat jejunum but could selectively block the activity of pancreatic lipase as a competitive inhibitor. These findings suggest that crocin has lipid lowering properties by inhibiting pancreatic lipase, leading to malabsorption of fat and cholesterol [44].

#### Pancreatic Lipase Inhibitory Activity of Saffron

Pancreatic lipase inhibitor has attracted much attention for its key role in obesity treatment due to its effectiveness and low toxicity. Orlistat has been a familiar pancreatic lipase inhibitor available in the market as an anti-obesity drug since 1999. In terms of inhibiting the dietary fat absorption, orlistat is more effective when compared with crocin (orlistat reduces fat absorption by approximately 30% at the dose of 40 µmol/kg [65] and crocin reduces fat absorption by 12% at the dose of 102 µmol/kg). Orlistat powerfully inhibits the activities of both gastric and pancreatic lipases, but crocin has higher selectivity for pancreatic lipase. The inhibition of orlistat on lipase is irreversible, whereas the inhibition of crocin is reversible [44]. Further crocin is fully unabsorbable while minimum orlistat could be absorbed, which may sometimes result in hepatotoxicity [44]. Usually, orlistat has some gastrointestinal side effects including oily spotting, flatulence and frequent loose stools [66]. However, crocin does not have those side effects and was confirmed to be nontoxic [67] which may be attributed to its moderately mild inhibition on lipase.

### 3.3. Hypoglycemic and Anti-Diabetic Effects of Saffron

Reportedly saffron significantly increased serum insulin and lowered blood glucose in diabetic rats [45]. Crocin was found to possess anti diabetic property in rodents fed fructose as it relieved free fatty acid induced insulin insensitivity and dysregulated mRNA expression of TNF-alpha, adiponectin as well as leptin in primary cultured rat adipocytes proposing the possibility of crocin prescription as a preventive approach of insulin resistance and the related diseases [54,68]. Advanced glycation end products are associated with the cause of oxidative reaction that normally occurs in endothelial cell apoptosis and thus results in diabetic vascular complications. Crocin by virtue of its good antioxidant capacity and calcium antagonistic action or stabilization may be a good solution for diabetic vascular complications [55]. El-Daly [69] described that *Crocus sativus* stigmas given together with cisplatin led to an even greater reduction in blood glucose than that seen with cisplatin.

#### Glucose Uptake Regulatory Effect of Saffron

Recently Kang *et al*. (2012) elucidated mechanism of the hypoglycemic actions of saffron through investigating its signaling pathways associated with glucose metabolism in C(2)C(12) skeletal muscle cells. They found that saffron strongly enhanced glucose uptake and the phosphorylation of AMPK (AMP-activated protein kinase)/ACC (acetyl-CoA carboxylase) and MAPKs (mitogen-activated protein kinases), but not PI 3-kinase (Phosphatidylinositol 3-kinase)/Akt. According to their results, the co-treatment of saffron and insulin further improved the insulin sensitivity via both insulin-independent (AMPK/ACC and MAPKs) and insulin-dependent (PI 3-kinase/Akt and mTOR) pathways. In line with the findings of GLUT4 translocation, it was also suggested that there is interference between the two signaling pathways of glucose metabolism in skeletal muscle cells. Overall, AMPK plays a key role in the effects of saffron on glucose uptake and insulin sensitivity in skeletal muscle cells [23].

### 3.4. Anti-Depressant and Mood Improving Effects of Saffron

Crocin and ethanolic extracts of saffron are known to show antidepressant impact on rodents. Crocin also reduced immobility time and increased climbing time at dose 50–600 mg/kg may be via individual uptake inhibition of dopamine and norepinephrine [48]. In another study, it was found that saffron supplementation statistically improved the moods of people compared with the placebo group after receiving 30 mg/day of saffron for six weeks evaluated based on the Hamilton Depression Rating Scale (HAM-D) [49]. A similar study by Noorbala and colleagues determined that saffron extracts were effective in treating mild to moderate depression similar to fluoxetine (the antidepressant, Prozac) after 30 mg/day intake for six weeks [51].

#### Satiety Enhancer and Weight Loss Promoter

Ethanolic extract of saffron stigma was found to significantly reduce the body weight in rats [27,51]. Decreased appetite has been shown as a clinical complication and side effect following the treatment with saffron [27,51]. In a human trial published in 2010, saffron by the name of *Satiereal* was under consideration as a satiety enhancer and weight loss promoter. In this study, mood-improving effect of saffron which would result in lowered appetite and snacking was investigated. Twice-daily, women subjects (*n* = 60, overweight) were given 1 capsule of *Satiereal* (176.5 mg/day) or an inactive placebo with no limitation in dietary intake. After 2 months, the subjects using the saffron extract reported a decrease in snacking and lost more weight than the control group [60]. Authors suggested that combination of an adequate diet with saffron supplementation as a *Satiereal* might help subjects engaged in a weight loss program in achieving their objective [60].

## 4. Link between Saffron’s Antioxidants and Possible Anti-Obesity Property

Obesity is a chronic disease of multi-factorial origin that develops from the interaction of social, psychological, behavioral, metabolic, cellular, and molecular factors [70]. It is the condition under which adipose tissue is increased and can be defined as an increase in body weight that results in excessive fat accumulation. The World Health Organization (WHO) defines obesity as a body mass index (BMI) > 30 and defines overweight as with a BMI of 25. In the last years, several studies have proposed that obesity might be an inflammatory disorder [71,72,73,74]. In addition, oxidative stress has been suggested as a potential inducer of inflammatory status and susceptibility to obesity and related disorders [70,71,75]. One possible strategy to reduce oxidative stress, inflammation and insulin resistance is consumption of antioxidant rich diet. A diet with high total antioxidant capacity has been found inversely related to central adiposity, metabolic and oxidative stress bio-markers, and risk for cardiovascular diseases [76,77,78]. Thus it is postulated that saffron directly or indirectly can inhibit obesity pathophysiology by working as an anti-inflammatory compound alone or fat reducing agent in parallel.

### 4.1. Mechanisms of Anti-Inflammatory Effect of Saffron

Potential mechanisms, by which saffron prevents obesity-mediated inflammation and related metabolic diseases, are still under investigation, but regarding its rich polyphenol/carotenoid content, saffron can assumedly reduce inflammation by (a) acting as antioxidant or increasing antioxidant gene or protein expression; (b) attenuating endoplasmic reticulum stress signaling; (c) blocking pro-inflammatory cytokines or endotoxin-mediated kinases and transcription factors related to metabolic syndrome; (d) suppressing inflammatory or inducing metabolic-gene expression via raising histone deacetylase activity; or (e) activating transcription factors that intensify chronic inflammation [38,79].

### 4.2. Mechanism of Potential Weight Loss Effect of Saffron

**Figure 2:**
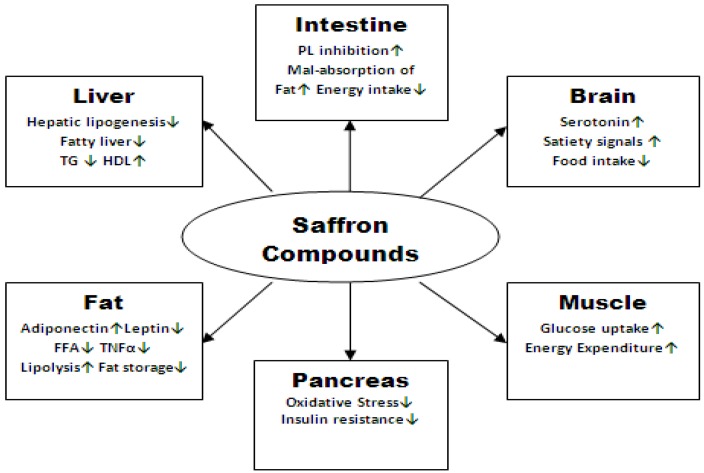
Schematic of possible anti-obesity effect of saffron compounds.

PL: Pancreatic lipase; TG: Triglyceride; HDL: High-density lipoprotein; FFA: Free fatty acid.

Although possible weight loss effect of saffron and its mechanism of action is not clear yet, saffron extract has a promising potential as an anti-obesity herbal medication through different biological functions which can be classified into four major categories including: (1) decreasing calorie intake by blocking dietary fat digestion via inhibiting pancreatic lipase; (2) acting as an antioxidant and suppressing inflammatory cytokines and adipocyte differentiation; (3) suppressing food intake by increasing satiety, or the feeling of fullness due to raising the level of neurotransmitters or hormonal functions; and (4) enhancing glucose and lipid metabolism all of which were briefly pointed out in section 3. Figure 2 shows a schematic of our postulation in potential anti-obesity effect of saffron based on the data from previous studies that were reviewed in section 3 earlier in the discussion on biological properties of saffron.

## 5. Conclusions

Antioxidant-rich saffron compounds may modulate obesity and associated metabolic disorders. They can be potentially useful in prevention, control, and/or management of overweight and obesity of individuals. Despite the lack of supporting evidence on possible weight loss effect of saffron in obese individuals, current knowledge about properties of saffron suggests that saffron supplementation will be at least responsible for lowering the risk of over snacking-diet associated with obesity or promoting weight loss in overweight individuals.

Due to lack of research on evaluating the efficacy of saffron as an anti-obesity medication and clarifying the possible mechanism of action, both pre-clinical and clinical studies are warranted to demonstrate its full health potential.
